# Supplementary figures and images for: Using topic modelling for unsupervised annotation of electronic health records to identify an outbreak of disease in UK dogs
Source: PLoS One. 2021 Dec 9;16(12):e0260402. doi: 10.1371/journal.pone.0260402 (PMC8659617; doi:10.1371/journal.pone.0260402)

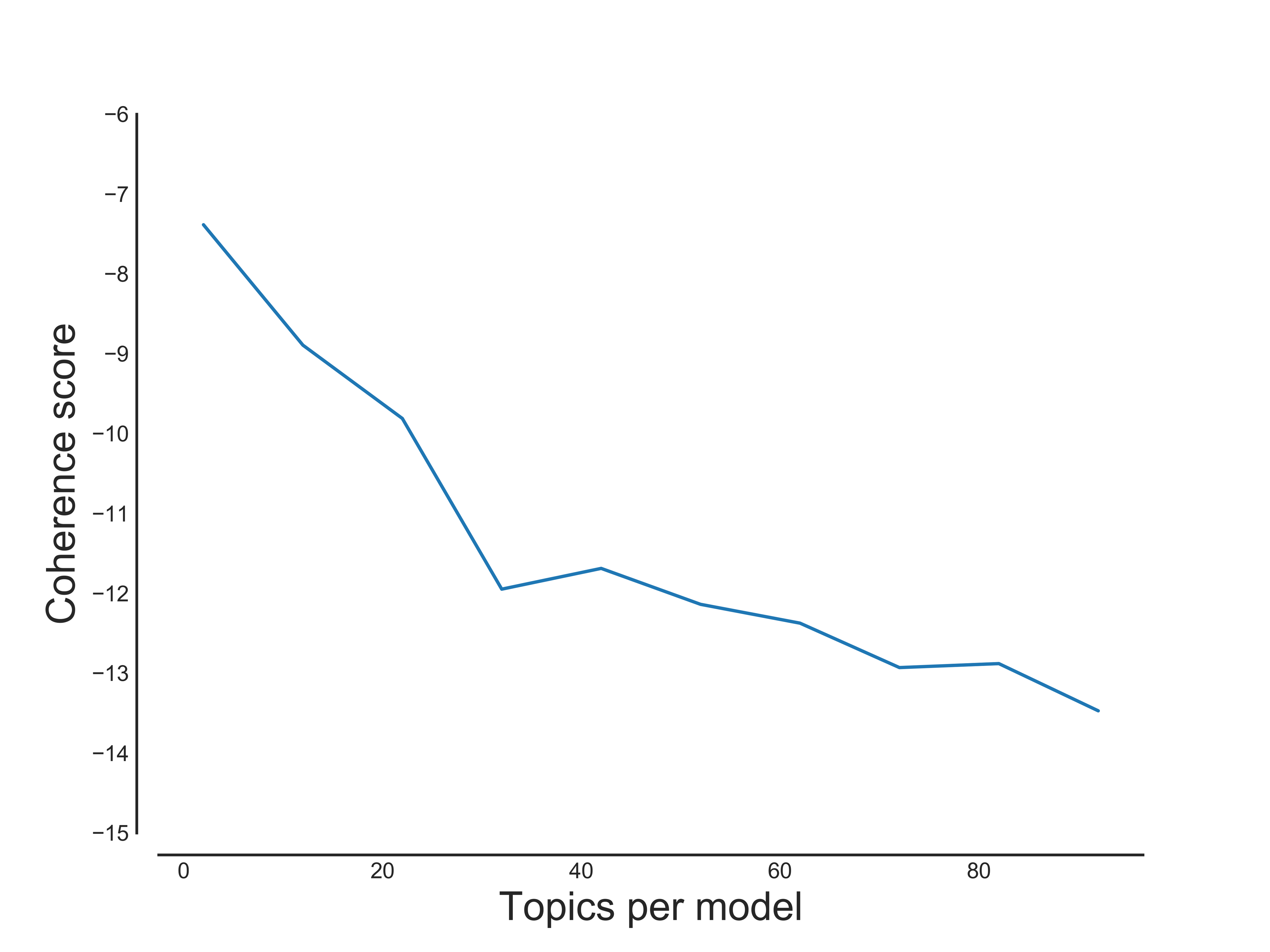

Supplement: S1 Fig — (TIF) [file pone.0260402.s001.tif]

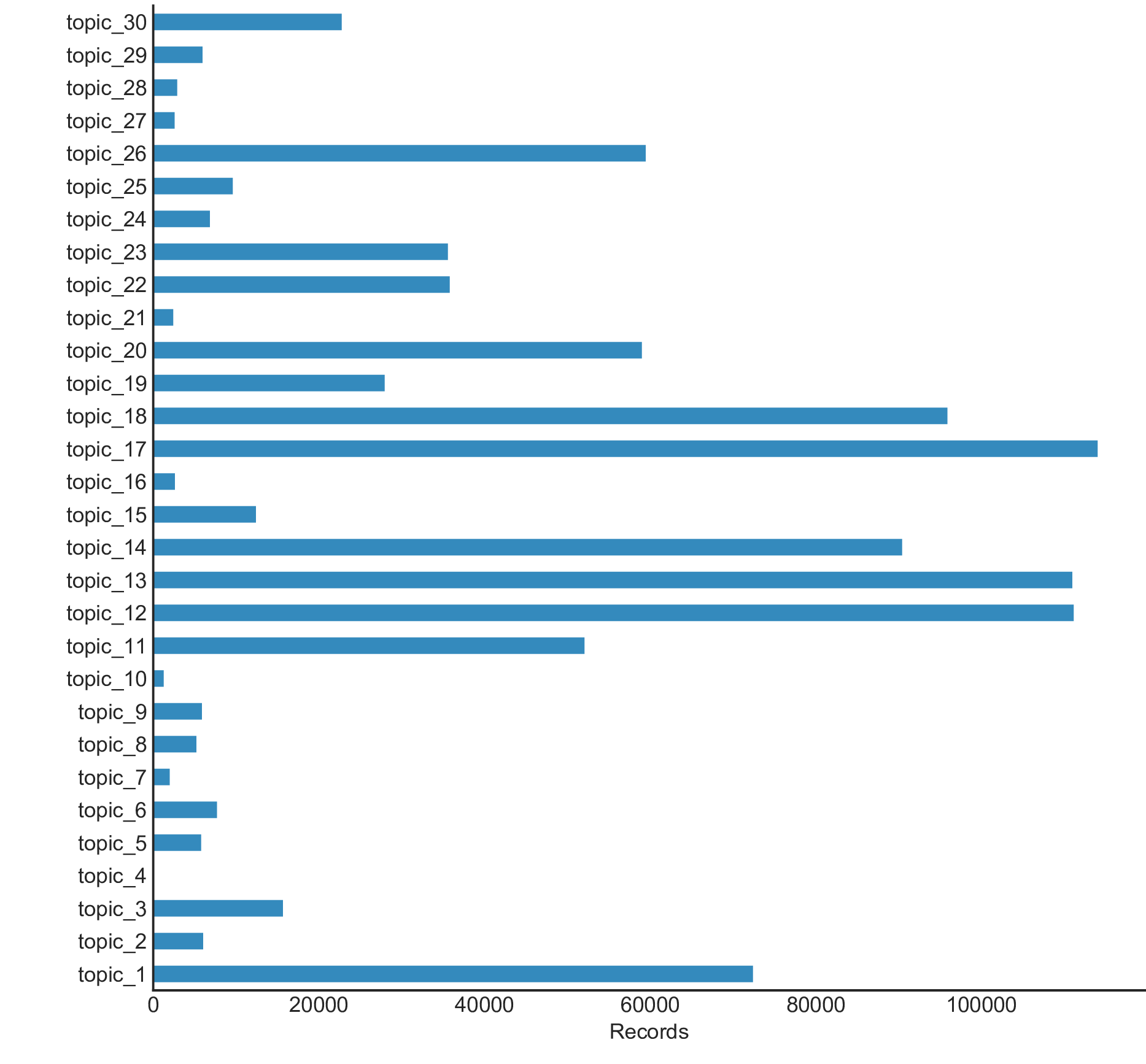

Supplement: S2 Fig — (TIF) [file pone.0260402.s002.tif]
