# Supplementary material for: Using topic modelling for unsupervised annotation of electronic health records to identify an outbreak of disease in UK dogs
Source: PLoS One. 2021 Dec 9;16(12):e0260402. doi: 10.1371/journal.pone.0260402 (PMC8659617; doi:10.1371/journal.pone.0260402)
Supplement: Attachment — Submitted filename: Response to reviewers.docx [file pone.0260402.s005.docx]

Friday 18.7.2021

Dear Editor,

Small Animal Teaching Hospital

University of Liverpool

Leahurst

Chester High Road

Neston

Wirral

CH64 7TE

Tel: +44 151 7956100

Fax: +44 151 7956101

Web: http://www.liv.ac.uk/sath

email: sath@liv.ac.uk

**Re: Original article submission entitled: “Using topic modelling for unsupervised annotation of electronic health records to identify an outbreak of disease in UK dogs.” – response to reviewers.**

Many thanks to the editorial team and to your reviewers for taking the time to look at this manuscript. I know that most of the folks who might review this paper will have had a mass of work on their hands due to COVID. Many thanks for the useful and constructive feedback from the reviewers.

I set out responses below:

## Academic editor comments

1. Please ensure that your manuscript meets PLOS ONE's style requirements, including those for file naming. The PLOS ONE style templates can be found at

<https://journals.plos.org/plosone/s/file?id=wjVg/PLOSOne_formatting_sample_main_body.pdf> and

<https://journals.plos.org/plosone/s/file?id=ba62/PLOSOne_formatting_sample_title_authors_affiliations.pdf>

Apologies for incorrect formatting, we have reviewed the document and hope we have corrected any deviations from the requested format

2. We noted in your submission details that a portion of your manuscript may have been presented or published elsewhere.

Please clarify whether this [conference proceeding or publication] was peer-reviewed and formally published. If this work was previously peer-reviewed and published, in the cover letter please provide the reason that this work does not constitute dual publication and should be included in the current manuscript.

Figure 6c is very similar to a figure in a peer reviewed publication (Radford et al 2021). In the current paper, this panel shows the trajectory of a disease outbreak which is described in the Radford et al paper. It is used as a comparison to the signal detected by topic modelling which is under discussion in the current paper. The Radford paper is primarily reporting the disease outbreak. In the current paper, we are illustrating how a novel method of automated record annotation could have mirrored the outbreak report in the Radford et al paper. We are not stating that we have identified the outbreak using the included figure, rather, that its inclusion clearly illustrates the utility of topic modelling to perform the same task.

3. We note that you have indicated that data from this study are available upon request. PLOS only allows data to be available upon request if there are legal or ethical restrictions on sharing data publicly. For information on unacceptable data access restrictions, please see <http://journals.plos.org/plosone/s/data-availability#loc-unacceptable-data-access-restrictions>.

In your revised cover letter, please address the following prompts:

a) If there are ethical or legal restrictions on sharing a de-identified data set, please explain them in detail (e.g., data contain potentially identifying or sensitive patient information) and who has imposed them (e.g., an ethics committee). Please also provide contact information for a data access committee, ethics committee, or other institutional body to which data requests may be sent.

SAVSNET data is collected in as anonymised form as possible, tabulated owner and pet names are not collected, neither are tabulated addresses or telephone numbers. However, the clinical free text data may include versions of these information. SAVSNET has automated redaction of such data to create deidentified text in the vast majority of cases, however, no deidentification software is 100% and some identifiers may get past this step, particularly unusual pet names which might identify the owner and as such the free text narratives are considered sensitive and are only shared through a data application process that commits users to appropriate curation of any data shared. The data access process is straightforward and can be initiated through e-mail approach to [savsnet@liverpool.ac.uk](mailto:savsnet@liverpool.ac.uk) or via our web-based data-access portal (<https://savsnetvet.liverpool.ac.uk/DAPP>). Further details of the data user agreement completed though this portal can be supplied if required for review.

b) If there are no restrictions, please upload the minimal anonymized data set necessary to replicate your study findings as either Supporting Information files or to a stable, public repository and provide us with the relevant URLs, DOIs, or accession numbers. Please see <http://www.bmj.com/content/340/bmj.c181.long> for guidelines on how to de-identify and prepare clinical data for publication. For a list of acceptable repositories, please see <http://journals.plos.org/plosone/s/data-availability#loc-recommended-repositories>.

We will update your Data Availability statement on your behalf to reflect the information you provide.

4. Thank you for stating the following in the Acknowledgments Section of your manuscript:

We note that you have provided funding information that is not currently declared in your Funding Statement. However, funding information should not appear in the Acknowledgments section or other areas of your manuscript. We will only publish funding information present in the Funding Statement section of the online submission form.

Please remove any funding-related text from the manuscript and let us know how you would like to update your Funding Statement. Currently, your Funding Statement reads as follows:

 [ADR, PJN, GN, CA  No funder reference number, DogsTrust, <https://www.dogstrust.org.uk>
ADR, PJN, GN BB/N019547/1, Biotechnology and Biological Sciences Research Council, <https://bbsrc.ukri.org>
ADR, PJN No funder reference number, British Small Animal Veterinary Association, <https://www.bsava.com>

The funders had no role in study design, data collection and analysis, decision to publish, or preparation of the manuscript.]

We have removed mentions of funders from the Acknowledgments section and the funding statement remains the same

Additionally, because some of your funding information pertains to [commercial funding//patents], we ask you to provide an updated Competing Interests statement, declaring all sources of commercial funding.

In your Competing Interests statement, please confirm that your commercial funding does not alter your adherence to PLOS ONE Editorial policies and criteria by including the following statement: "This does not alter our adherence to PLOS ONE policies on sharing data and materials.” as detailed online in our guide for authors  [http://journals.plos.org/plosone/s/competing-interests](about:blank).  If this statement is not true and your adherence to PLOS policies on sharing data and materials is altered, please explain how.

*Competing Interests statement*

We have not received commercial funding for this project. we presume the concern is that data contributors include commercial companies. In this case the companies include VetSolutions and Teleos whose practice management systems are modified to allow SAVSNET data collection and CVS who are corporate group of vet-practices from whom we collect data. None of these groups provide funding for the project or direct trajectory of research or publication and as such we have removed the word ‘support’ from the acknowledgement section. Please let us know if this requires further clarification.

Please include the updated Competing Interests Statement and Funding Statement in your cover letter. We will change the online submission form on your behalf.

Please know it is PLOS ONE policy for corresponding authors to declare, on behalf of all authors, all potential competing interests for the purposes of transparency. PLOS defines a competing interest as anything that interferes with, or could reasonably be perceived as interfering with, the full and objective presentation, peer review, editorial decision-making, or publication of research or non-research articles submitted to one of the journals. Competing interests can be financial or non-financial, professional, or personal. Competing interests can arise in relationship to an organization or another person. Please follow this link to our website for more details on competing interests: [http://journals.plos.org/plosone/s/competing-interests](about:blank)

5. Please include captions for your Supporting Information files at the end of your manuscript, and update any in-text citations to match accordingly. Please see our Supporting Information guidelines for more information: [http://journals.plos.org/plosone/s/supporting-information](about:blank).

Figure 2 and Figure 3a have been converted to supporting information as advised by reviewer#1 and along with captions fro these captions for supporting information data-files have been appended to the manuscript.

## Reviewers Comments -Reviewer #1:

The authors used topic model to mine text data to mine veterinary clinical narratives. They find that one of the topic mixture probabilities (topic 17) over time are indicative of gastroenteric presenting complaint (MPC). Using Bayesian binomial linear model, they confirm that the finding is statistically significant. The study show that it is possible to use topic model to detect outbreak of novel disease. Although there is no methodological innovations in topic modeling and the analyses are standard topic model analysis, it is a novel application and an interesting angle that the authors took in looking into the outbreak using topic models.

Major comments:
1. The authors were modeling temporal changes of the text data using LDA from the standard Gensim Library. Authors should use dynamic topic model instead to capture the changes of topics over time.

The main aim of our paper was to explore the feasibility of using topic modelling to identify a potential outbreak. For that, and as a first step, static models were considered a reasonable approach. Indeed, the signal identified using the ‘static’ model is demonstrated to be useful by its close mapping to the other outbreak metric (MPC). Still, following the reviewer’s comment, we have added to the paper how a dynamic topic model can be used to allow observers to infer potential evolution of the content of a given topic over time. We are currently evaluating a variety of other dynamic topic modelling approaches and topic modelling using tools such as BERTopic and Tomotopy, but we thought that a systematic evaluation of these is outside the scope of the current manuscript.

2. Evaluating only on the topic coherence in choosing the topic number is not sufficient because it only evaluates each topic separately not across topics. You can have coherent topics but the topics can all look the same. A proper metric is called topic quality that combines topic coherent and topic diversity (Mimno et al 2011).

This is a fair comment. However, for the purpose of monitoring for emergence of a syndrome signalling an outbreak, review of the topic contents would quickly identify ‘vacuous’ topics (we have added mention of these to the manuscript) meaning that the model can err on the side of too many topics while still being useful (as it was in this case).

3. Authors mention computational overhead caused by choosing more topics. This is possibly because they were using the full-batch LDA training on 3.5 million records in their data. LDA can be inferred by stochastic variational inference using minibatch of the records (also known as online LDA). This should be implemented in the Gensim Library.

We have opted to use a smaller subset to experiment with the number of topics, which is now hopefully clearly explained in the manuscript. Computational overheads were not the main limiting factor in building the model given the number of topics.

4. I am confused on whether the model was trained on real data and then applied to the simulated data or trained and applied directly on the simulated data. It seems that the former is true. I would argument that the latter is more realistic because in real world application, you can’t train on text in the absence of the outbreak at least not in the same time interval.

This is now clarified in the manuscript.

5. Figure 2 and 3a are in very poor quality. A more compact display is needed. Otherwise, Figure 2 and 3a should be in supplementary.

Figure 2 has been removed with explanation in the text. Figure 3a has been replaced with a figure illustrating clustering of topics with MPCs which illustrates the topic-mpc relationship more helpfully

Minor comments:
6. The flow and the format of the paper makes it sometimes hard to follow. In particular, in Results section, figure legends were inserted in the middle of the text with large margin around them (e.g., page 14).

The authors accept that the placement of figure legends does disrupt the text but figure legends were placed in accordance with editorial guidelines(https://journals.plos.org/plosone/s/figures):

“Place figure captions in the manuscript text in read order, immediately following the paragraph where the figure is first cited. Do not include captions as part of the figure files or submit them in a separate document.”

7. The authors call the veterinary clinical narratives of dogs as electronic health records (EHR). I wonder to what extent this is the case. There is no standardized data such lab tests LOINC or RxNorm prescription.

We indeed have access to EHR (or EPR), which contain some structured data (species, breed, age, sex), prescription data and clinical narrative. While the data comes from various providers and is not standardized to any particular standard, it still does represent EHR. However, we have made it clearer in text that we only use the narrative part.

## Reviewer comments - Reviewer #2:

The objective of the study was well motivated as to the use of topic modelling to classify EHR into categories that would allow for surveillance. What was not well explained is the criteria and definition of disease outbreak. The point was made that the utility of labelling would be to facilitate detection of outbreaks. When in the development of the outbreak should the system be making the decision that an outbreak is present? If it waits until the outbreak is at its worst then obviously this would mean that there would be little utility for prevention or control. On lines 186-8, an outbreak is equated to an increase in gastroenteric MPC but that is a very vague definition and does not take into account many different sources of variation (dog show in town, new clinic hours, holidays, etc.). These sources of variation are what makes syndromic surveillance so difficult because of the problem of false positives.

This paper seeks to illustrate that an unsupervised method can produce results comparable to supervised annotation of clinical records in the context of a disease outbreak. The exact mechanism for alerting the presence of an outbreak from this data remains to be decided and is being researched by the SAVSNET team. We have added a paragraph to the discussion alluding to this citing an elegant publication that offers a means to address this.

It was also mentioned that there is a great deal of uncertainty in topic modelling with regards to negatives - this is indeed a problem and I did not clearly see any explanations for how this was mitigated in this study.

In a sense the problem of negation is ignored in the face of a signal that tracks gastroenteric disease regardless of how the topic model emerges from the data. We do not address this in the discussion because the topic model does allow identification of the outbreak regardless of how negation of those words occurs in the original texts

The other large issue with natural language data sets is misspellings, short forms, etc. From my own experience with human hospital and veterinary clinic records points to a huge variation of spellings unless the doctor/vet is constrained or there is post-processing to correct spellings or expand abbreviations (which is only possible for a known set of abbreviation variations). I did not see where it was explained about the processing of the data from the EHR and since I am not familiar with the UK systems for this type of data collection I am confused as to whether this is actually an issue.

The authors fully agree with this sentiment (having mis-spelled and abbreviated many a clinical narrative) but in effect, the topic modelling deals with this by treating all words/abbreviations/acronyms as tokens which comprise a topic agnostic of what those are. We have added detail to how tokens were generated in the methods.

I was also unclear as to what the effect was if the EHR was describing more than one condition of interest. It is also unclear if the reason that this topic modelling works well is because GI has mainly 2 symptoms (vomiting and diarrhoea).

Most documents were given a greater than zero probability for more than one topic and this vector of topic probabilities will be studied in further work. For the purpose of this paper, the authors felt that the key message that an unsupervised annotation method could identify an outbreak scenario which succeeded while only looking at a single topic annotation using the topic for which a narrative had a >0.5 probability. We have added a note in the discussion regarding further, more comprehensive analysis of narrative-topic probabilities.

It was briefly discussed with respect to the overlap between cardiac and respiratory symptoms and diagnoses but it does leave the reader waiting for a subsequent study that looks at this question.

That study is on the way alongside use of newer topic modelling techniques that articulate word-meaning more effectively and therefore create more meaningful topics

I would caution against justifying the choice of topics (30) because of computational cost without putting this into perspective. In other words, what type of computational system was used and how long did it take to produce results.

We have added detail regarding the modest computational capacity used for this study and hope that this spurs other investigators to consider this type of work even when high performance computing is not available.

I did find the use of unsupervised methods appropriate and useful for this task and think that this study is an excellent first step in a more comprehensive use of this technique.

Many thanks, we feel this is very much a first step and, hopefully, one of many

Yours sincerely,

P-J.M.Noble BSc BVM&S PhD FHEA MRCVS
